# Correlative light and electron microscopy of poly(ʟ-lactic acid) spherulites for fast morphological measurements using a convolutional neural network

**DOI:** 10.1093/jmicro/dfab058

**Published:** 2021-12-22

**Authors:** Yuji Konyuba, Hironori Marubayashi, Tomohiro Haruta, Hiroshi Jinnai

**Affiliations:** EM Business Unit, JEOL Ltd., 3-1-2 Musashino, Akishima, Tokyo 196-8558, Japan; Institute of Multidisciplinary Research for Advanced Materials, Tohoku University, 2-1-1 Katahira, Aoba-ku, Sendai, Miyagi 980-8577, Japan; Application Management Department. JEOL Ltd., 3-1-2 Musashino, Akishima, Tokyo 196-8558, Japan; Institute of Multidisciplinary Research for Advanced Materials, Tohoku University, 2-1-1 Katahira, Aoba-ku, Sendai, Miyagi 980-8577, Japan

**Keywords:** poly(ʟ-lactic acid), correlative light and electron microscopy, transmission electron microscopy, polarized optical microscopy, spherulite, convolutional neural network

## Abstract

Polarized optical microscopy (POM) and transmission electron microscopy (TEM) are widely used for imaging polymer spherulite structures. TEM provides nanometer resolution to image small spherulites of sub-micrometer in diameter, while POM is more suitable for observing large spherulites. However, high-resolution images with a large field of view are challenging to achieve due to the deformations of ultrathin sectioned samples used for TEM observations. In this study, we demonstrated that correlative light and electron microscopy (CLEM) combining POM and TEM could effectively characterize the spherulite structures in a wide range from nanometer to several hundred micrometers that neither TEM nor POM alone could cover. Furthermore, the deformations of the TEM ultrathin sections were corrected by referencing to the POM images at the same position of the sample, and large-area TEM images without deformations were successfully produced. The spherulite structures of poly(ʟ-lactic acid) were successfully analyzed using CLEM and TEM in a wide range of spatial scales at the same field of view. The large-area TEM image (250 µm × 250 µm), consisting of 702 TEM images stitched together, was subjected to machine learning to extract the essential structural information of spherulites. Analysis using the convolutional neural network, a well-known algorithm You Only Look Once (YOLO), demonstrated that it was practical to accurately obtain the diameter distribution and the space-filling factor (relative crystallinity) of the spherulites. This study presents a new approach for acquiring high-resolution images with a large field of view and processing the images at a fast speed.

## Introduction

Crystalline polymers, such as polyethylene, are used in a wide range of applications because of their lightweight, low cost and excellent processability. The physical properties of crystalline polymers largely depend on their hierarchical solid-state structures with sizes spanning the nanometer to micrometer range [1–4]. For example, the smaller the size of the spherulites (spherical assemblies of lamellar crystals on the order of 1–100 μm), the better the transparency of the molded product. Therefore, understanding the hierarchical structure is essential for the structural design of crystalline polymeric materials. For this reason, it is desirable to have a method to analyze each level of the hierarchical structure at various scales.

Polarized optical microscopy (POM) has often been used to observe spherulites because the molecular orientation in the spherulite can be observed from the change in polarization state when linearly polarized light is incident on the sample. In addition, by combining POM with temperature control equipment, it is possible to observe the growth of spherulites (crystallization behavior) by melting crystalline polymers and then cooling them to their crystallization temperatures [5]. However, the resolution of POM is limited to a feature size of ∼250 nm, making it difficult to detect small spherulites on a submicron scale. The limitation of POM can be complemented using the electron microscopy, such as transmission electron microscopy (TEM). Various studies on the crystalline morphologies of polymers using TEM and POM have been reported [6–11]. Furthermore, systems, such as correlative light and electron microscopy (CLEM) [12–16], have been developed that combine a light microscope and an electron microscope (i.e. TEM), which allows the simultaneous imaging of samples using these two methods.

However, there are two possible challenges in using CLEM with POM and TEM. The first challenge is the sample deformation during microtoming. In ultrathin sectioning using an ultramicrotome, soft material samples may be deformed by the stress during slicing. Such deformation causes a mismatch between TEM and POM images and makes it difficult to obtain high-quality images with the CLEM. The direction and amount of deformation of the TEM sample can be estimated by comparing large-area TEM and POM images. Therefore, the TEM image can be corrected by superimposing it on the POM image. The second challenge is the limitation in the field of view. TEM samples are usually supported by metal grids in which the distance between grid bars is a few tens of microns. Therefore, if the same field of view as POM (e.g. several hundreds of microns square) is imaged by TEM, a part of the sample information will be covered by grid bars. To obtain large-area TEM images without grids, the use of a chip with a large-area freestanding silicon nitride sample support film [17–19] has been proposed. Thereafter, an image of a large field of view with a high-resolution can be produced by stitching TEM images together.

The correlative POM and TEM would provide valuable information on the hierarchical structures of polymers, such as the number density of spherulites, including those with small diameters (nucleation density), and the percentage of space occupied by spherulites (space-filling factor) within the desired region. To detect spherulites from TEM images and analyze their properties (i.e. size, diameter and area), a reliable method is needed to prevent human errors and speed up the analyses. An automatic detection algorithm using machine learning is one such method with high reliability and reproducibility. In addition, it has a much higher throughput than manual work and can analyze large amounts of image data in a short time. Such object detection methods have made significant progress in the last few years and have been applied to TEM [20–22].

In the present study, we investigated poly(ʟ-lactic acid) (PLLA), a crystalline polymer that has recently attracted attention as an environmentally friendly polymer derived from plants [23] using a CLEM system. We successfully overlaid the high-resolution TEM images onto the POM images using deformation correction. To match the field of view of the TEM and POM images, a large number of TEM images were stitched together to produce a high-resolution large-area TEM image. A convolutional neural network-based object detection system was applied to the large-area TEM image to detect each spherulite automatically. Finally, the diameter distribution and space-filling factor (relative crystallinity) of the spherulites within the desired region were calculated from the results of the spherulite detection.

## Methods

### Sample preparation

The PLLA (-[-OCH(CH_3_)CO-]*_n_*-) used in this study (Lacty 5408, Toyota Motor Corporation, Japan) has the number average molecular weight (*M_n_*) = 6.0 × 10^4^ g/mol, polydispersity index (*Ð*) = 1.8 and specific rotation $ \left[ \alpha \right]_{\rm{D}}^{25} =$ −155 deg· dm^−1^· g^−1^· ml (chloroform), which is similar to that of the optically pure PLLA ($\left[ \alpha \right]_{\rm{D}}^{25} =$ −156 deg· dm^−1^· g^−1^· ml [24]). The pellets of PLLA were purified by reprecipitation (*M_n_*, *Ð* and specific rotation $\left[ \alpha \right]_{\rm{D}}^{25}$ were measured after reprecipitation), and small sample pieces were sandwiched between a glass slide and a cover glass. The sample was heated at 200°C for 2 min to melt, cooled to the crystallization temperature *T_c_* (120°C) at 10°C/min and then isothermally annealed at *T_c_* for 1 min to crystallize. Thereafter, the sample was allowed to cool naturally to room temperature (approximately 24°C). The thickness of the obtained film was 13 ± 2 μm.

### Set up for POM observation

The spherulites of PLLA at *T_c_* were observed using a digital microscope (VHX-600, KEYENCE Corp., Japan) equipped with a temperature-controlled stage (LTS350, Linkam Scientific Instruments Ltd., UK). POM observation was conducted in a crossed Nicols optical system with a sensitive color plate.

### Sample preparation for TEM

A film of PLLA on a glass slide was glued to the tip of an epoxy resin block (Epon812) and trimmed into a pyramid shape to match the imaging field of the POM observation. The PLLA was then sliced to a thickness of 200 nm using an ultramicrotome (Leica UC6, Leica Microsystems, Germany), and continuous ultrathin sections were mounted on a chip with a SiN sample support film [25] (SiN window chip, JEOL Ltd., Japan). To reduce the damage caused by electron beams, the surface of the sample was coated with a 10-nm-thick carbon film using a carbon coater (CADE-4T; Meiwafosis Co., Ltd., Japan).

### Set up for TEM observation

A 120-kV transmission electron microscope (JEM-1400Flash with Limitless Panorama; JEOL Ltd., Japan) equipped with an automatic montage system was used to acquire large-area TEM images. Observation conditions for each captured TEM image are listed as follows: number of pixels = 2048 × 2048 and pixel size on the sample plane = 17.8 × 17.8 nm^2^. The imaging area was the entire ultrathin section (approximately 470 μm × 620 μm), and 702 TEM images (27 vertical and 26 horizontal) were stitched together to produce a large-area TEM image. The procedure for image alignment using Limitless Panorama involves the following steps: (i) Calculate the amount of misalignment using normalized cross-correlation for adjacent images taken in a tile pattern. (ii) When adjacent images are aligned in sequence, distortions may occur. For example, when the image of the first row is aligned and subsequently the image of the second row is aligned, inconsistency will be present between the rows. Here, the final position of the image is determined using the least-squares method to minimize the error due to the adjacent movement of the group of images connected by the alignment.

### Overlay of POM and TEM images

The same field of view as the POM image was extracted from the acquired large-area TEM image, and the POM and TEM images were overlaid. The images were aligned using Fiji [26] and SightX viewer (JEOL Ltd., Japan). The spherulites and bubbles in both images were used as fiducial marks. Using the distance between the two fiducial markers, the deformation coefficient of the TEM image was calculated using the following equation.

$$\begin{align*}& {\rm{Compensating\ deformation\ factor\ for\ TEM\ image}} = \\ & \quad{{{\rm{Distance\ between\ bubbles\ in\ POM\ image}}} \over {{\rm{Distance\ between\ bubbles\ in\ TEM\ image}}}}\end{align*}$$

### Detection of spherulites and calculation of space-filling factor

To detect spherulites, we used YOLOv5 [27] from the YOLO (You Only Look Once) series [28,29], a well-known object detection system using convolutional neural networks. Among the dataset comprising 263 TEM images (1024 px × 1024 px for each image) containing spherulites of PLLA, 213 images were used as the training set, and 50 images were used as the test set. LabelImg [30] was used to annotate the images. The training was performed on a Quadro P4000 (Nvidia Corporation, Santa Clara, CA, USA) with 8 GB memory in the Anaconda virtual environment. The training process took about 11 h. The convolutional neural network generated by the training was used to detect the locations, widths and heights of the spherulites in the large-area TEM image. The width and height of each spherulite were used to calculate its area, assuming that the shape of the spherulite was an ellipse. Finally, the area calculated for each spherulite was summed up and divided by the area of the image to calculate the space-filling factor of the spherulites.

## Results and discussion

### Correlation between POM and TEM

**Fig. 1.**
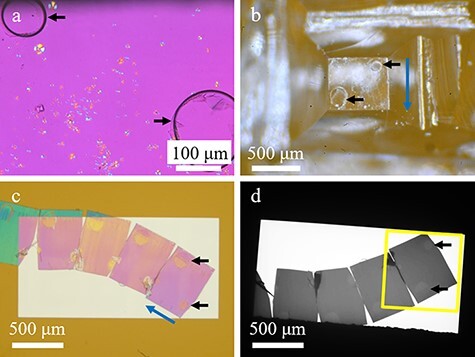
Various microscopic images of the PLLA sample. Black arrows indicate bubbles. Blue arrows indicate ultramicrotome cutting direction. (a) POM image. (b) PLLA film-mounted resin block after trimming. Image is mirror-symmetric to the image in (a) because this image was taken from the illumination direction. (c) Optical microscopy image of the SiN window chip after mounting a ribbon of ultrathin sections. (d) Low-magnification TEM image. The yellow frame indicates the imaging range of the large-area TEM image.

**Fig. 2.**
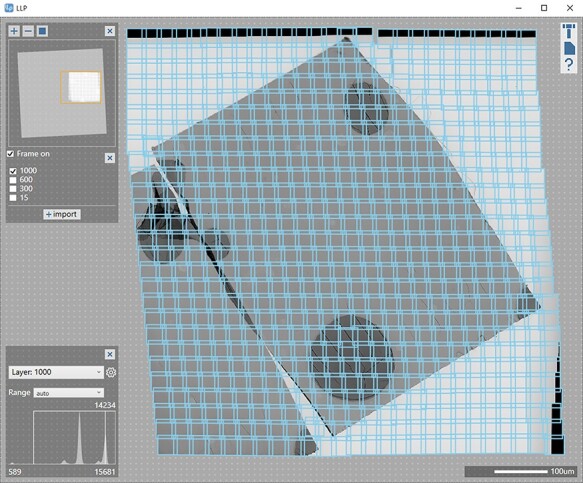
Results of large-area TEM observation, showing the field of view within the yellow frame in Fig. 1d. Each blue frame indicates an individual TEM image, all of which were pieced together by Limitless Panorama to create this image.

The POM image of the isothermally crystallized PLLA is shown in Fig. 1a. There were spherulites of various sizes that have grown radially. At 120°C for 1 min, the degree of crystallinity was still low, and there were many regions (purple areas) where no spherulites were observed. In some areas, isolated spherulites continued to grow, while small spherulites had already collided in other areas. The birefringence properties of each spherulite were also confirmed as color changes. Yellow color in the first and third quadrants and blue color in the second and fourth quadrants indicated the negative birefringence. The molecular chains (*c*-axis) were oriented perpendicularly to the radius of the spherulites in the interior of the lamellar crystal growing radially from the center [31]. Using the two bubbles (indicated by arrows in Fig. 1a) in the POM image as markers, we trimmed and prepared ultrathin sections (Fig. 1b and c) and confirmed the presence of two bubbles in the low-magnification TEM image (Fig. 1d). The results of the large-area TEM observation of the entire ultrathin section are shown in Fig. 2. We generated a single image by stitching together 702 TEM images that were not damaged or deformed by electron irradiation.

**Fig. 3.**
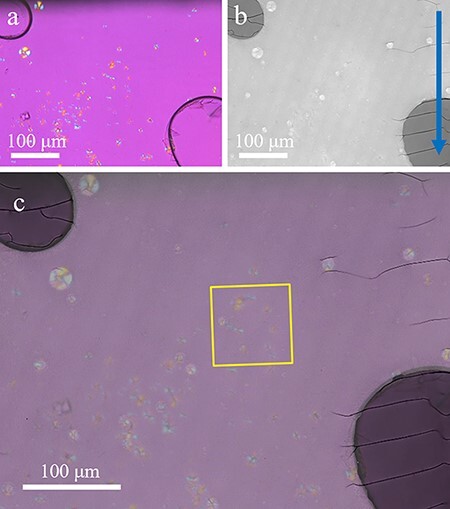
Application of CLEM to PLLA by POM and TEM. (a) POM image. (b) Large-area TEM image of the same field of view as the POM image (deformations caused during ultrathin section preparation have been corrected). Blue arrow indicates the cutting direction during the preparation of ultrathin sections. (c) Merged image of the POM and TEM images. The yellow frame indicates the area shown in Fig. 4.

By overlaying the POM image (Fig. 3a) and the large-area TEM image (Fig. 3b), it was confirmed that they coincided when the TEM image was scaled perpendicularly to the cutting direction of microtoming by approximately 0.86. Therefore, the elasticity/plasticity and shear stress of PLLA correspond to the stretching ratio between the POM and TEM images. As the strain in TEM image is less than 1%, it can be ignored as a nonlinear element. Figure 3c is a merged image of the POM and TEM images in which the two images overlap with overlay error of approximately 1 μm. These results showed that the ultrathin sections prepared in this study were stretched only in the direction perpendicular to the cutting direction, and the deformation was about 1.16 (=1/0.86) times.

**Fig. 4.**
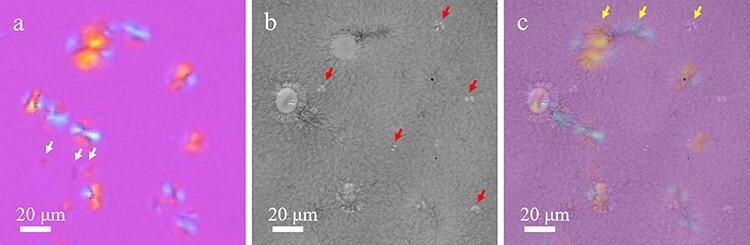
Difference between the spherulites in the POM and TEM images. Each image represents the enlarged view of the yellow-framed region in Fig. 3c. (a) POM image. White arrows indicate the spherulites that are not observed in the TEM image. (b) Large-area TEM image (corrected for deformation caused during ultrathin section preparation). Red arrows indicate the spherulites that are not observed in the POM image. (c) Merged image of the POM and TEM images. Yellow arrows indicate spherulites shown in Fig. 5.

Figure 4 shows the enlarged images of the yellow-framed region in Fig. 3c. In Fig. 4b, the spherulites marked with red arrows are visible only in TEM images [not visible in POM image (Fig. 4a)]. This was likely due to the fact that the sizes of the spherulites were smaller than the POM resolution (∼250 nm). In contrast, in Fig. 4a, the spherulites marked with white arrows are visible only in POM [not visible in TEM (Fig. 4b)]. This was probably because they were positioned either above or below the TEM section along the film thickness direction. Note that the thickness of the sample for the POM observation was 13 μm, from which the thin section of 200-nm thickness was microtomed.

**Fig. 5.**
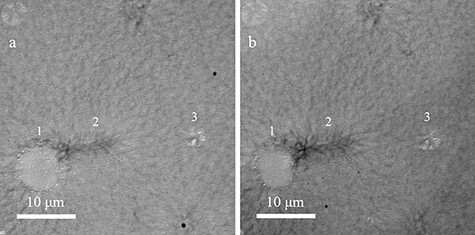
Comparison of the spherulite shape between adjacent ultrathin sections. Each image is an enlargement of the area containing the spherulites indicated by the yellow arrows in Fig. 4c, with the same field of view. Deformations caused during the preparation of ultrathin sections have been corrected. (a) TEM image extracted from the large-area TEM image. (b) TEM image of the same field of view as (a), from an ultrathin section that has been advanced by 200 nm in the depth direction.

Figure 5a shows the enlarged TEM image containing the spherulites indicated by the yellow arrows in Fig. 4c. Figure 5b shows the TEM image of the adjacent ultrathin section obtained by serial sectioning. By comparing the same spherulites in Fig. 5a and b (as indicated by the numbers), it was found that the shape of the spherulite changed (spherulite 1 became smaller; 2 and 3 became larger). The reason was that the diameters of the spherulites were much larger than the section thickness (200 nm) and the spherulite shape in the TEM images represented the projected area of the spherulites in the ultrathin-sectioned films. The shape of the spherulite changed in different sections because the size of the projected area varied along the direction that was perpendicular to the ultrathin sections.

### Detection of spherulites and calculation of space-filling factor by convolutional neural network

**Fig. 6.**
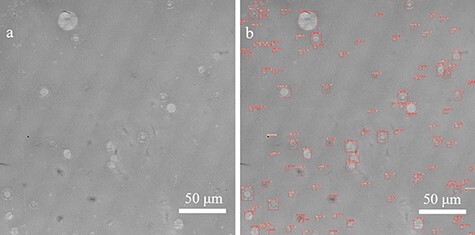
Results of spherulite detection by the convolutional neural network. (a) Image of a 250 μm × 250 μm area from the large-area TEM image. (b) TEM image after spherulite detection. Each red frame shows a detected spherulite and its width and height.

**Fig. 7.**
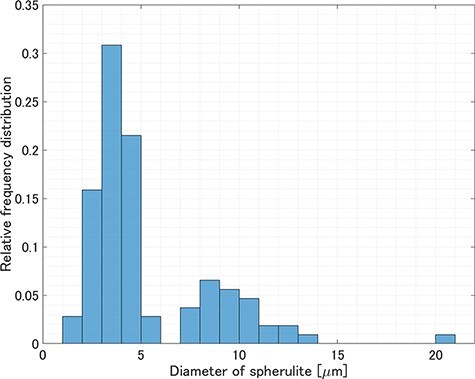
Relative frequency distribution of the diameter of spherulites detected in a 250 μm × 250 μm area of the large-area TEM image.

**Fig. 8.**
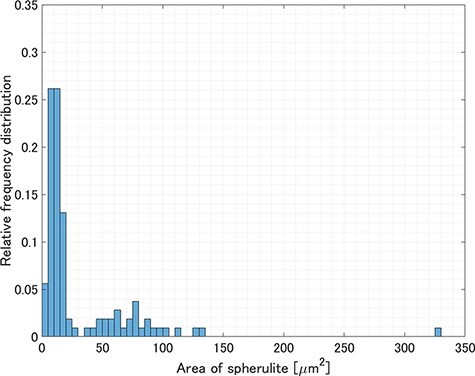
Relative frequency distribution of the area of spherulites detected in a 250 μm × 250 μm area of the large-area TEM image.

Figure 6b shows the results of spherulite detection using a convolutional neural network on a 250 μm × 250 μm region (Fig. 6a) extracted from the large-area TEM image (Fig. 2). The detection process took approximately 3 s. Although most of the spherulites were successfully detected, few spherulites could not be detected because the contrast difference between the background and the spherulites was low and the shape was not fully included in the training set. The successful detection factor was 81%. The number of spherulites detected in the 250 μm × 250 μm area was 107, and hence the average number of the detected spherulites per unit area was 0.0017 μm^−2^. Figure 7 shows the relative frequency distribution calculated from the diameter of each detected spherulite. The spherulites with a diameter smaller than 6 μm accounted for about 74%, while those with a larger diameter than 6 μm accounted for the remaining 26%. The minimum diameter of the detected spherulites was 1.6 μm. Figure 8 shows the relative frequency distribution calculated from the area of each detected spherulite. It was found that spherulites with an area smaller than 20 μm^2^ (corresponding to a spherulite with a diameter of 5 μm, if the cross-section of the spherulite is a perfect circle) accounted for about 71%, while those with an area larger than 20 μm^2^ accounted for the remaining 29%. The space-filling factor of the spherulites in this region was 5.2%. Because crystalline and amorphous regions coexist inside the spherulites [32,33], the space-filling factor of the spherulites calculated here represented a relative value, not an absolute value of crystallinity. The space-filling factor including the undetected spherulites was 6.3%. Approximately equal values were obtained when the space-filling factor, calculated from the detected spherulites, was corrected using the detection factor (5.2%/0.81 = 6.4%). The space-filling factor was 8.3%, calculated by manually extracting spherulites from POM images of the same region and assuming the shape of all spherulites to be spherical. This difference is observed because POM can extract the averaged molecular orientation information of spherulites, but it cannot yield the thickness information of spherulites. This method can be used to estimate the space-filling factor of spherulites (relative crystallinity) within a desired region, i.e. the spatial distribution of crystallinity in a sample, which cannot be obtained by traditional methods, such as calorimetry that measures the crystallinity of bulk samples. Furthermore, when combined with serial section TEM using continuous ultrathin sections [34,35], it is possible to analyze hierarchical structures in three dimensions.

## Conclusion

Using POM and TEM, the spherulitic structure of PLLA was observed in the same field of view. We have successfully produced a TEM image having a field of view as large as that of POM using a total of 702 TEM images stitched together. By overlaying the images acquired from both microscopes, it was found that the microtomed sample for TEM was stretched about 1.16 times in the direction perpendicular to the cutting direction due to stress during the preparation of ultrathin sections. POM can detect spherulites in non-deformed samples which can be used for correcting the large-area TEM images, while TEM can detect spherulites at a resolution much higher than that of POM. Therefore, the combination of both microscopes in the CLEM system made it possible to obtain large-area TEM images without the concern of sample deformation and enabled us to observe tiny spherulites that POM was not able to detect.

We proposed a method to detect spherulites from the acquired large-area TEM image using YOLO, a well-known object detection system using convolutional neural networks, and obtained the number density and space-filling factor (relative crystallinity) of spherulites within the desired region. Such local structural information is key to understanding the nucleation and growth mechanisms of polymer spherulites. This detection process is extremely fast and much more powerful than manual processing and therefore is expected to analyze large amounts of image data. Furthermore, the present method would be more effective than the conventional methods in analyzing the heterogeneously distributed spherulites in materials (e.g. preferential nucleation at the filler surface in polymer composites).
